# Unsupervised Indoor Localization Based on Smartphone Sensors, iBeacon and Wi-Fi

**DOI:** 10.3390/s18051378

**Published:** 2018-04-28

**Authors:** Jing Chen, Yi Zhang, Wei Xue

**Affiliations:** 1 Jiangnan University, School of Internet of Thing Engineering, Wuxi 214122, China; 6151910050@vip.jiangnan.edu.cn (Y.Z.); 6161914038@vip.jiangnan.edu.cn (W.X.); 2 Engineering Research Center of Internet of Things Technology Applications, Ministry of Education, Wuxi 214122, China

**Keywords:** indoor localization, iBeacon, initial localization, reliable model, fingerprint database

## Abstract

In this paper, we propose UILoc, an unsupervised indoor localization scheme that uses a combination of smartphone sensors, iBeacons and Wi-Fi fingerprints for reliable and accurate indoor localization with zero labor cost. Firstly, compared with the fingerprint-based method, the UILoc system can build a fingerprint database automatically without any site survey and the database will be applied in the fingerprint localization algorithm. Secondly, since the initial position is vital to the system, UILoc will provide the basic location estimation through the pedestrian dead reckoning (PDR) method. To provide accurate initial localization, this paper proposes an initial localization module, a weighted fusion algorithm combined with a k-nearest neighbors (KNN) algorithm and a least squares algorithm. In UILoc, we have also designed a reliable model to reduce the landmark correction error. Experimental results show that the UILoc can provide accurate positioning, the average localization error is about 1.1 m in the steady state, and the maximum error is 2.77 m.

## 1. Introduction

Indoor localization has gained a lot of attention for its wide application since the development of various methods for localization such as Wi-Fi [1,2], Radio Frequency Identification (RFID) [3], and so on. However, Wi-Fi is restricted by its fixed power and indoor environment and RFID needs corresponding equipment. Bluetooth low energy (BLE) has risen to prominence suddenly in the last two years for its low energy consumption and low cost [4,5,6]. iBeacon, a kind of equipment based on BLE, inherits the advantages of BLE which makes it much better than Wi-Fi in terms of energy consumption and cost. Furthermore, the deployment of iBeacon is much easier than RFID and Wi-Fi. Thus, iBeacon can set up a network for indoor localization easily.

The received signal strength (RSS) of Wi-Fi is frequently used for localization nowadays. Fingerprinting is a RSS-based localization and has been the most accurate approach to indoor localization among other RSS-based techniques [7], since it can help solve the problem of multipath and the non-line-of-sight (NLOS) propagation. However, the fingerprinting approach is labour intensive, and requires significant work to collect the RSS.

Given the shortcoming of the fingerprinting approach above, many papers [8,9,10,11] have recently put forward the idea of constructing the fingerprint by crowdsourcing. The database is updated by accepting the feedback from the user. However, this idea needs large quantities of feedback information to ensure its accuracy, so cooperation is important. The feedback from users may also contain mistakes. Therefore, the stability of crowdsourcing is low.

Another widely adopted localization technique is pedestrian dead reckoning (PDR) [12]. This includes the following modules: step detection, step length and walking direction. However, PDR has the problem of accumulative error and cannot estimate the initial point.

Some research and papers [5,13,14,15,16] use landmarks to correct a user’s location, but these landmarks cannot ensure whether the user is in the landmarks, they can only guess that the user is close to the landmarks. So the system always tries to haul back to the location of the landmarks at an unnecessary time.

In this paper, we propose UILoc, a new framework of combining PDR, iBeacon and Wi-Fi. In the PDR approach, since relative information is employed, this will drift with walking distance. Therefore, this paper intends to employ iBeacon measurements to correct the drift of the PDR approach occasionally. A fusion algorithm is applied to estimate the initial position, and the fingerprint database auto-building module is designed to improve the accuracy of the fusion algorithm. The reliable model is designed to provide a reliable location estimation and reduce the scope for error during the landmark rectification phase.

The rest of the paper is organized as follows. Section 2 introduces some related work about different localization methods. A system overview is presented in Section 3. Section 4 shows the detail of the UILoc. The prototype for implementation and experiments are discussed in Section 5. We conclude the work in Section 6.

## 2. Related Works

### 2.1. Trilateration Method

In the outdoor environment, trilateration is a common method for calculating the position of an unknown node. In the field of indoor localization, the trilateration method is often used to calculate the approximate position of the target node, which may be at room-level localization accuracy. This method requires at least three source nodes with known locations. In addition, the distances between these source nodes and target nodes also need to be known. According to [4,17], in the wireless network environment distance relationships can be converted from the corresponding signal strength values. Theoretically, each source node forms a circle around itself with the radius of the distance to the target node, and the point of intersection M is the target node. However, due to the fluctuation of wireless signals, the three circles cannot intersect at one point. In general, the optimal solution can be found by the least square method [18].

### 2.2. Fingerprinting-Based Localization

Since the early 20th century, fingerprint localization technology has been a common positioning method. In 2000, Microsoft proposed the RADAR system [1,19], and used the nearest neighbor algorithm and K-nearest neighbor algorithm for indoor localization first. The Nibble [20] system proposed by the University of California, Los Angeles, used a Bayesian probability model. In 2005, Youssef et al. of the University of Maryland proposed a Horus [21] algorithm based on a signal histogram probability model, which stores the probability distribution of the received signal strength indicator (RSSI) signals in a fingerprint database. Later, Youssef et al. made some improvements on the basis of the original Horus [22]. This algorithm mainly added a cluster partitioning module, signal compensation mechanisms, and an incremental triangle algorithm. Moghtadaiee et al. improved the K nearest neighbor algorithm and proposed the k-weighted nearest neighbors (KWNN) [23,24], that combined the weighted K nearest neighbors and Bayesian probability methods, making the positioning results significantly improved.

### 2.3. Pedestrian Dead Reckoning (PDR)

In recent years, with the popularization of smart phones, an increasing number of sensors have been integrated into smart phones. The smartphone can sense more environmental data, which further promotes the development of indoor-positioning technology. The PDR method utilizes inertial sensory data (including acceleration data and direction data) to estimate the relative instead of the absolute position. In the paper [25], Jin et al. showed that the main concerns of PDR technology are: the step detection algorithm, the walking length estimation, and the walking direction estimation. Wang et al. proposed the AD-FSM [26] algorithm, which improved the multi-state finite state machine. This algorithm can accurately count steps in a noisy environment. In UPTIME [27], the step-size estimation and walking direction estimation are described in detail. Kang et al. proposed SmartPDR [12] after combining gyroscope data and magnetometer data. The PDR method has good reliability over a short period. However, if there is no other reference, the PDR method may have serious cumulative error problems [9].

### 2.4. Hybrid Localization Method

In order to improve the accuracy and stability of the positioning algorithm, it is necessary to exploit the advantages of each positioning method while avoiding possible disadvantages. Many hybrid positioning algorithms have been proposed recently. Wang et al. proposed UnLoc [28], which uses the Wi-Fi fingerprint data of a specific location, the acceleration characteristics of stairs or elevators, and the magnetic characteristics of special locations in the environment as landmark points. The problem of cumulative error in PDR can be corrected at these landmark locations. In 2015, Yang Fan et al. of Shanghai Jiaotong University proposed a positioning method [29] combining PDR and Wi-Fi, in which the estimated position of the PDR can be used to reduce the fingerprint database matching range of the fingerprint method.

At the 2013 Apple Developers Conference, Apple Inc. proposed iBeacon technology based on BLE 4.0. Because of its small size, easy deployment and low power consumption, it has been widely used in indoor positioning in recent years. In [30,31], iBeacon-based indoor positioning methods are proposed. Specifically, in 2015, Chen et al. [5] combined the trilateration positioning and PDR methods, using a particle filter (PF) to fuse these algorithms. The particle propagation model in this algorithm is based on the PDR method. Then, in the correction phase, the results of the trilateration method are used as observations. In 2017, Jenny et al. also proposed a hybrid positioning method based on the Kalman method [32], and results show that this hybrid method can provide highly stable localization accuracy of less than one meter.

## 3. Summary of UILoc System

**Figure 1:**
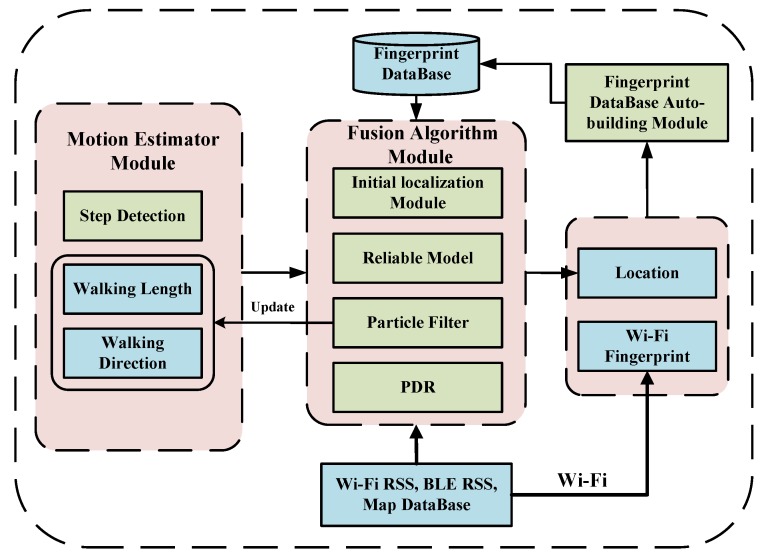
Overview of UILoc system.

The system architecture of the UILoc is shown in Figure 1, which mainly consists of a PDR module, particle filter module, reliable model (RM) module, fingerprint database auto-building module and initial localization module. Compared to fingerprint approaches, the UILoc builds the fingerprint database automatically in the online phase, which is similar to Zee [9], instead of sampling through considerable labour in the offline phase. In UILoc, the location is calculated by PDR. A particle filter module is used in PDR to correct the walking length and walking direction. Because the PDR method needs the initial position, the UILoc uses an initial localization module to provide the initial position. The UILoc uses the reliable model module to provide an accurate correction service to solve the problem of accumulated error. Then, the UILoc combines position information and fingerprint information to establish the fingerprint database which serves for the initial localization module to improve the initial positioning accuracy.

### 3.1. PDR Module

In the PDR approach, the current position is calculated from the previous position and can be expressed as follows:

(1) $$X_{t+1}=X_{t}+L_{t}\left[\begin{array}{c} sin\theta_{t}\\ cos\theta_{t} \end{array}\right],$$

where $X_{t}$ is the 2D location of the pedestrian, $L_{t}$ is the walking length, and $\theta_{t}$ is the walking direction at the time of step t. Some critical issues need to be addressed, including walking length estimation and walking direction estimation.

In this paper, we used the AD-FSM algorithm [26] to detect the step, because the AD-FSM algorithm can restrain noise interference effectively and provide stable and accurate step detection. The walking direction and walking length are corrected by a particle filter which will be introduced in the following section.

### 3.2. Particle Filter Module

In this paper, a particle filter [33,34] is used in walking length and walking direction estimation. As every particle has attributes of step length and walking direction deviation, the UILoc can correct the step length and walking direction.

**Figure 2:**
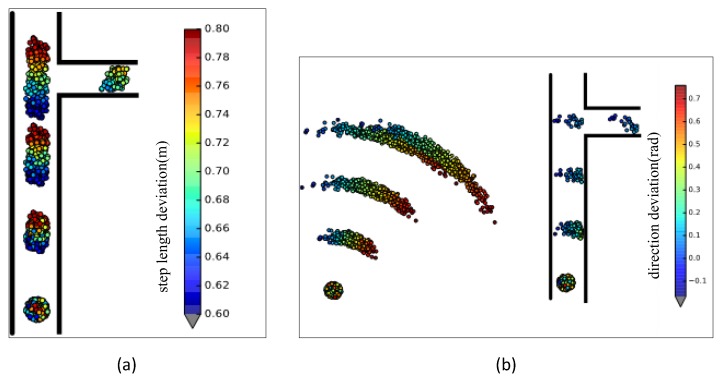
The scenario using particle filter. (**a**) The mechanism to correct the step length. (**b**) The mechanism to correct the walking direction.

Step length and walking direction deviation corrected by practical filtering are shown in Figure 2. In the initial phase, all particles are initialized at the initial position as a Gaussian distribution. Every particle has the attribute of step length bias and walking direction bias. With the user’s movement, the positions of the particles will be different, and many erroneous particles will die because they hit the wall. The mean of step length deviation and walking direction deviation of the remaining particles are the user’s real walk state.

### 3.3. Reliable Model Module

The reliable model is proposed to provide reliable location estimation and reduce the error scope during the landmark rectification phase. This model will be described in the next section.

### 3.4. Fingerprint Database Auto-building Module

The fingerprint database auto-building module combines the position after fusion localization and the fingerprint to update the fingerprint database. This module is designed to serve for the initial localization module.

### 3.5. Initial Localization Module

The initial position is very important for the whole localization because it influences the position error. The initial localization module combines the fingerprint approach and propagation model localization. It can provide the accurate initial position.

The details for each individual subsystem are introduced in the following sections.

## 4. Proposed Method 

### 4.1. Fingerprint Database Auto-Building Module

The fingerprint database auto-building module builds and updates the Wi-Fi fingerprint database automatically in an unsupervised state. The position information is mainly provided by the fusion algorithm module. The UILoc adopts this module for a more accurate initial position.

The fingerprint database has no data in the initial phase. At the beginning, the positioning of the system depends mainly on the propagation model of iBeacon. Then, UILoc can estimate the position of the user by PDR combined with the particle filter. Also, the UILoc deploys some iBeacons as landmark points in the indoor environment for correcting the cumulative error caused by PDR. With the continuous construction of the fingerprint database, the initial location provided by the initial localization module that fuses the fingerprint and propagation model method will become increasingly accurate.

The main process of building the fingerprint database is as follows:

1. Firstly, since we can get the location (${\mathrm{loc}}_{\mathrm{online}}$) and Wi-Fi information through this system at the online phase, we need to find the fingerprint in the fingerprint database whose location can be expressed as ${\mathrm{loc}}_{\mathrm{db}}$, and the distance between these two points need to be less than 1 m. Otherwise, we insert the information into the fingerprint database directly.
2. Since we can find the fingerprint in the database, then the position and value of RSSI in the fingerprint will be averaged, and update the database.

### 4.2. Initial Localization Module

This paper proposes an initial localization module to reduce the error of the initial position. Because there is no fingerprint database in the initial phase, the approach commonly used in iBeacon-based position is the propagation model method. Most users focus on the location of the first localization when they use the positioning service, and initial localization is very important to the localization system. Although the deviation of the initial localization is large when the fingerprint database in the initial phase is not complete, with the completion of the fingerprint database the localization system based on the fingerprint can provide a more accurate initial position. A new method which combines the propagation and fingerprint methods is proposed to improve accuracy. This algorithm is briefly explained in the following subsections.

**Figure 3:**
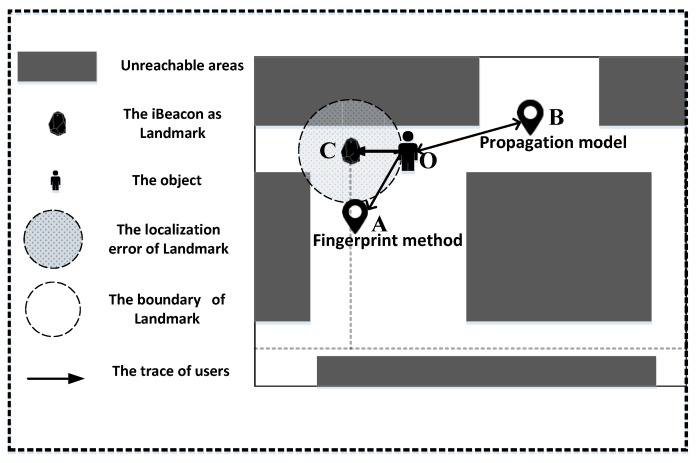
The principle of the initial localization module.

As shown in Figure 3, when a user requests localization at the point O, the UILoc system will scan all signals in the environment first. Then, the signal strength of each iBeacon will be transformed into distance. The relationship between RSS value and distance is obtained by polynomial fitting [4].

The mapping relations between RSS data and distance in our environment is defined as follows:

(2) $$Distance=0.568*rssi+0.0079*rssi^{2}+10.2$$

Since a smartphone has the data of the user’s orientation and the system has all the positions of landmarks (iBeacons), we can find the landmark that has the strongest RSS. Then, the initial localization module uses the landmark position and orientation to infer the possible position of the user.

According to the KNN calculation formula, we calculate the position of A. The formula is defined as follows:

(3) $$D_{j}=\sqrt{\sum_{i=1}^{n}{\left(RSS_{online}\left(i\right)-RSS_{j}\left(i\right)\right)}^{2}},$$

where $D_{j}$ represent the Euclidean distance of the *j*th reference point (RP), and i is the number of the access point (AP).

In the nearest neighbors(NN) algorithm, the device is estimated at the most similar measuring point (i.e., with the minimal Euclidean distance). The KNN computes the center of k’s closest neighbors and uses Euclidean distance as the weight of each neighbor. The formula is defined as follows:

(4) $$Loc_{KNN}=\frac{1}{K}\overset{k}{\underset{i=1}{\sum }}loc_{i}$$

Of course, through the least squares method we can also estimate the position. The specific formula is as follows: we assume that the user’s location is O(x,y) and iBeacons that can be detected by the user are located at $\left(x_{i},y_{i}\right)$, so the distances between user and iBeacons can be expressed as:

(5) $$r_{i}{}^{2}={\left(x_{i}-x\right)}^{2}+{\left(y_{i}-y\right)}^{2},$$

(6) $$r_{i}{}^{2}=K_{i}-2x_{i}x-2y_{i}y+x^{2}+y^{2}\left(i=1,2,\ldots ,N\right),$$

where $K_{i}=x_{i}{}^{2}+y_{i}{}^{2}$.

We define $r_{i,1}$ as follows:

(7) $$r_{i,1}=r_{i}-r_{1}$$

(8) $$r_{1}=r_{i|i=1}=\sqrt{{\left(x_{1}-x\right)}^{2}+{\left(y_{1}-y\right)}^{2}}$$

So we can get:

(9) $$r_{i}{}^{2}=r_{i,1}{}^{2}+2r_{i,1}r_{1}+r_{1}{}^{2}$$

Combining Equation (6) with Equation (9), we can get:

(10) $$r_{i,1}{}^{2}+2r_{i,1}r_{1}=-2x_{i,1}x-2y_{i,1}y+K_{i}-K_{1},$$

where $x_{i,1}$ and $y_{i,1}$ represent $x_{i}-x_{1}$ and $y_{i}-y_{1}$. Note that the unknowns $r_{1},x$ and y have a linear relation. So this can be expressed as a matrix form:

(11) $$\frac{1}{2}\left[r_{i,1}{}^{2}-\left(K_{i}-K_{1}\right)\right]=-\left(r_{i,1}r_{1}+x_{i,1}x+y_{i,1}y\right)$$

(12) $$-\left[\begin{array}{ccc} x_{2,1} & y_{2,1} & r_{2,1}\\ x_{3,1} & y_{3,1} & r_{3,1}\\ \vdots & \vdots & \vdots \\ x_{N,1} & y_{N,1} & r_{N,1} \end{array}\right]\left[\begin{array}{c} x\\ y\\ r_{1} \end{array}\right]=\frac{1}{2}[\begin{array}{c} r_{2,1}{}^{2}-\left(K_{i}-K_{1}\right)\\ r_{3,1}{}^{2}-\left(K_{i}-K_{1}\right)\\ \vdots \\ r_{N,1}{}^{2}-\left(K_{i}-K_{1}\right) \end{array}]$$

This can be expressed as:

(13) GZ=H

So the location of user is calculated as:

(14) $$Loc_{LS}=Z={\left(G^{T}G\right)}^{-1}G^{T}H$$

Then, the fusion algorithm is expressed as follows:

(15) Dfp=1edisKNN,

(16) Dls=1edisLS,

where $dis_{KNN}$ represents the distance of |OA|, and $dis_{LS}$ represents the distance of |OB|. Since the larger distance indicates the greater error, we use the exponent of e to amplify the deviation, and then get the reciprocal of it because the greater of the error, the smaller the weight to be given. Then, the weights need to be normalized as follows:

(17) $$W_{fp}=\frac{D_{fp}}{D_{fp}+D_{ls}}$$

(18) $$W_{ls}=\frac{D_{ls}}{D_{fp}+D_{ls}}$$

The final position after normalizing the weight is estimated as follows:

(19) $$Loc_{out}=W_{fp}*Loc_{KNN}+W_{ls}*Loc_{LS}$$

### 4.3. Reliable Model

The reliable model module can shrink the error produced by the landmarks’ correction and provide stable and accurate localization. We propose the reliable model to provide a reliable location estimation and reduce the scope of error during the landmark rectification phase.

**Figure 4:**
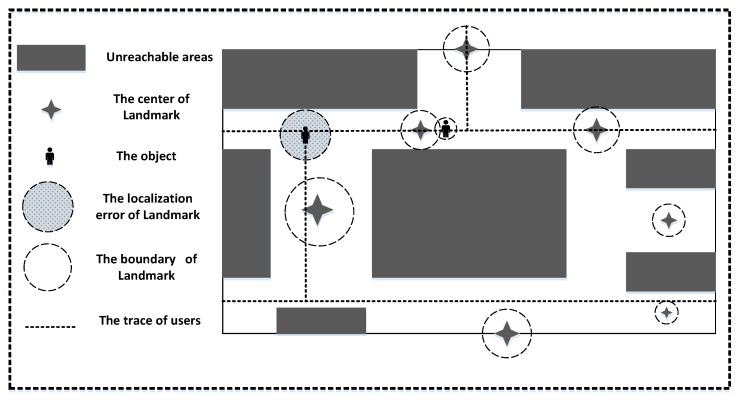
The scenario using landmarks.

Figure 4 depicts the scenario using landmarks in the indoor environment. There are many landmarks which can correct users’ locations in the inner area of a building, but the identify-boundary of a landmark may be a rough scope whose position cannot be measured precisely. The landmark cannot make corrections efficiently when the user is keeping close to the landmark because of the fluctuation of the BLE signal. Landmarks may correct the error caused by PDR, but the error from itself still remains. So our aim is to minimize the error from landmarks.

Most papers [5,14] use this method to detect the strongest signal. When the algorithm finds that the value of RSS is greater than the threshold, the user’s location will pull back to the center point of a landmark, but at that time the user may just come close to the landmark area or has left the landmark area.

**Figure 5:**
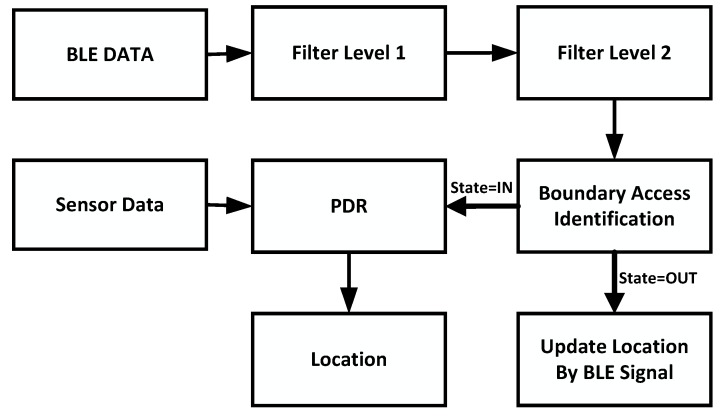
The design of the reliable model.

To smooth the signal and restrain noise, we designed a reliable model that can improve the location accuracy. As Figure 5 shows, the algorithm consists of the boundary access identification module, L1 and L2 filter, and the PDR module. Firstly, the L1 level filter is utilized to eliminate the outliers. Then, the L2 level filter is used to restrain noise and smooth the signal. Next, the boundary access identification module judges the states of the users about specific landmarks; if a user has not entered the landmark area, the user’s location will be corrected by the landmark and the location will be produced by PDR. Lastly, once the user has accessed the reliable model, the PDR module will provide the output of localization.

**Figure 6:**
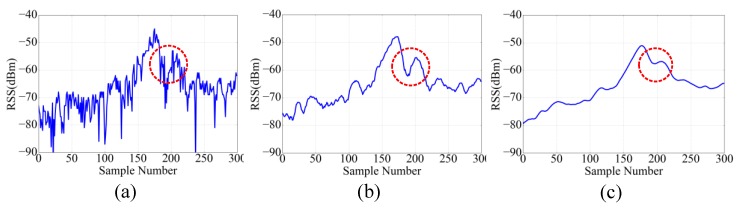
The signal curves of the iBeacon when the user comes close to then goes away from the landmark. (**a**) Raw data of bluetooth low energy (BLE). (**b**) Data of BLE after L1 filter. (**c**) data of BLE after L2 filter.

Figure 6a shows the process whereby the user comes close to the landmark then goes away from it. Because of the fluctuation of BLE RSS, shown as the red circle labeled in the figure, at that time the user has already gone away from the landmark, but the value of RSS is larger than the threshold again, so the algorithm will correct the user’s position. In this case, the raw data will enhance the volatility and lower the robustness in the process of localization.

Firstly, we considered the sampling frequency is 10 Hz, so the length of the window for the dynamic filter was set to 10. The L1 level filter is designed as below:

(20) $$\{\begin{array}{l} RSS=w\left(rss_{i-9},rss_{i-8},\ldots ,rss_{i}\right)\\ RSS^{\prime }=filter\left(RSS\right)\\ rss_{i}=\frac{1}{n}\sum_{j=1}^{n}rss_{j} \end{array},$$

where RSS is a dynamic vector about the value of rss. We used Tukey’s test method to remove the outliers for RSS and get $RSS^{\prime }$. Finally, the mean value of $RSS^{\prime }$ which will be given to $rss_{i}$ is calculated, where $rss_{i}$ represents the rss at the moment i. The Tukey’s test method is expressed as follows:

(21) $$\{\begin{array}{l} min=Q_{1}-1.5\left(Q_{3}-Q_{1}\right)\\ max=Q_{3}+1.5\left(Q_{3}-Q_{1}\right)\\ min<rss_{k}<max \end{array},$$

where $Q_{1}$ and $Q_{3}$ are expressed in Equation (22), and each reasonable $rss_{k}$ in RSS must be between the  min and the max.

(22) {Q1=sorted(RSS)14∗length(RSS)Q3=sorted(RSS)34∗length(RSS)

After the L1 level filter, the signal curve is shown in Figure 6b. It is clear that there is a lot of Gaussian noise which will influence the localization, so we proposed to use the Kalman filter to eliminate the Gaussian noise. The algorithm contains two processes: predicting and updating.

Firstly, the Kalman filter has some parameters that have to be determined. As the value of RSSI changes randomly, therefore, the transition matrix F and the measurement matrix H are set to one. Furthermore, there is no external control input, so $Bu_{t-1}$ is also set to zero.

Predicting:

(23) $$\{\begin{array}{l} rss_{t|t-1}=Frss_{t-1|t-1}+Bu_{t-1}\\ P_{t|t-1}=P_{t-1|t-1}+Q_{t-1} \end{array}$$

Updating:

(24) $$\{\begin{array}{l} K_{t}=P_{t|t-1}H_{t}^{T}{\left(H_{t}P_{t|t-1}H_{t}^{T}+R_{t}\right)}^{-1}\\ rss_{t|t}=rss_{t|t-1}+K_{t}\left(rssi_{t}-H_{t}rss_{t|t-1}\right)\\ P_{t|t}=\left(1-K_{t}H_{t}\right)P_{t|t-1} \end{array}$$

In Equation (24), P is the estimate covariance. In UILoc, the initial value of P is set to $\;P_{t-1|t-1}=1.0$. K is the Kalman gain. The values Q=0.015 and R=1.5 are determined experimentally.

After the L1 and L2 level filter, the signal curve is shown in Figure 6c.

The basic principles of the reliable model are described in detail above. Algorithm 1 is the pseudo-code form of the reliable model.

 **Algorithm 1** Reliable Model

**Input:** *ble_data, ori*

**Output:** *location*

**Loop:**
*ble_data_L1* = filteredByL1(*ble_data*)*ble_data_L2* = filteredByL2(*ble_data_L1*)//boundary access identification module start*beacon_i* = find the beacon whose state is *IN***if***beacon_i_state***is***IN***and***beacon_i_rssi* < −65: *beacon_i_state = OUT**beacon_max* = find the beacon which has max rssi**if***beacon_max_state***is***OUT***and***beacon_max_rssi* > −52: *distance* = **map** *beacon_max_rssi* **to** *diatance* X = beacon_max_postion_x − distance * sin(ori) Y = beacon_max_postion_y − distance * cos(ori) *beacon_max_state = IN* **return** *X,Y*
**End Loop**



The input data of the reliable model are the BLE signal $\left\{UUID_{i}:RSSI_{i}\right\}$ and the direction data in the environment. The output is the location of the user. Once the reliable model gets the data, the BLE data will be filtered by L1 and L2. We then need to set the states of users about specific landmarks from IN to OUT when the RSSI from a specific beacon is less than −65 dBm (about 6 m by Equation (2)). At that moment, we can think that the user has gone out of the landmark area completely. Next, we need to find the beacon as beacon_i which has the strongest signal. If the state of beacon_i is OUT and the strength of beacon_i is greater than −52 dBm (about 2 m by Equation (2)), we then need to update the user’s location as follows. We also need to set the state of beacon_i to IN, so even if the user walks into the landmark area, the reliable model can prevent the problem of repeated positioning. In the landmark area, the location will be calculated by PDR.

### 4.4. UILoc Algorithm Explanation

All the sub modules have been described in detail above. Next, we will describe the details of the module integration. Algorithm 2 is the pseudo-code form of the UILoc.

 **Algorithm 2** UILoc

**Input:** *data*

**Output:** *location*

**Loop:**
use *data* to update *acc, ori, wifi_entry*, *ble_entry* and *ble_queue_window* by *data’s*
type**if***type***is***ACC*: *acc_filtered* = use kalman filter to filter *acc* **if** *AD-FSM.countStep(acc_filtered)* **and** *location* **is** not null:  *location* = use particle filter to estimate the *location***else if***type***is***BLE*: **if** *location* **is** not null:  *location* = *ReliableFilter.process(ble_queue_window,ori)*
**else if** *type* **is** *WIFI*
 **if** *location* **is** null:  *location = InitLocalization(wifi_entry,ble_entry)*  **initialize** the particle filter module **else:**  **update** fingerprint database by *location* and wifi_*entry*
**return** *location*

**End Loop**



The input of the UILoc is data {type:senor_data}, including acceleration data, orientation data, BLE data and Wi-Fi data. The output of the algorithm is the location of the user.

Firstly, the parameter ble_entry is the data like $\left\{UUID_{i}:RSSI_{i}\right\}$. The parameter wifi_entry is a fingerprint data like $\left\{MAC_{i}:RSSI_{i}\right\}$ and the parameter ble_queue_window is an array that contains 10 historical ble data.

If the type of data are  ACC, we do a Kalman filter for acc data, then we use AD-FSM to determine whether a step is formed. If the location has been initialized and the acc data formed a step, then the location will be calculated by the particle filter which has fused the PDR module. If the data are a type of  BLE, we use the reliable model to provide a stable localization. Lastly, if the data are a type of  WIFI, we use initial localization module to estimate the user’s initial location. We can also create and update the fingerprint database.

## 5. Experimental Work and Results

In this section, we introduce the experimental setup, which was used to perform the proposed model in a real building (i.e., other people may walk through this area during the experiments), and then present and discuss the experimental results.

### 5.1. Experimental Setup

**Figure 7:**
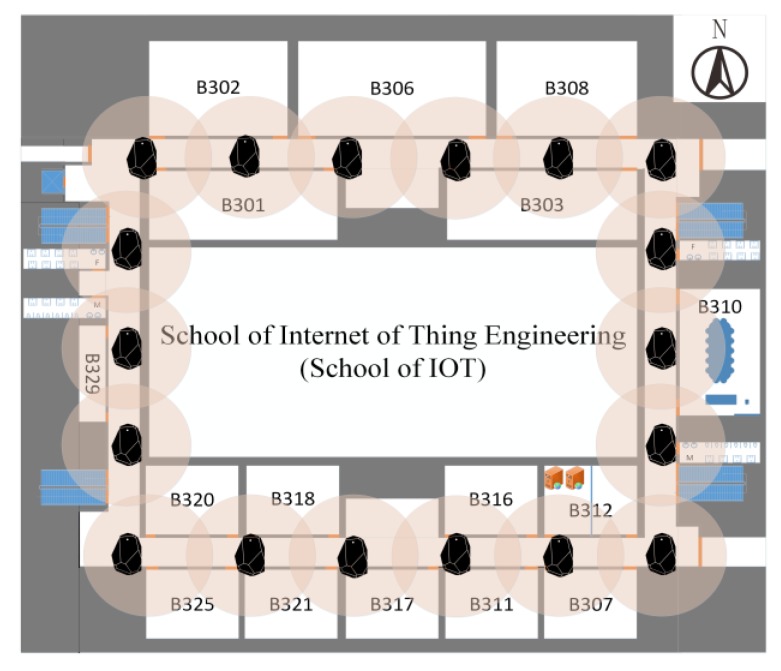
Layout of the experimental environment.

To evaluate the performance of the UILoc model, we conducted an experiment on one floor of a typical office building at the campus of JNU (Jiangnan University). The size of the target area is about 3000 $m^{2}$ (60 m by 50 m). Figure 7 shows the layout of the research lab. We deployed 18 beacons (average: 1 beacon per 10 m) in the experiment. All of these beacons were installed on the wall at a height of approximately 1.5 m. To balance the power consumption and accuracy, each beacon was set to a 10 Hz sample rate with 0 dBm transmit power. The device involved in the experiment is a HUAWEI P9 smartphone running an Android 6.0 operation system. In order to compare the fingerprint localization algorithm, we performed our experiment with 184 RPs. The average distance between 2 RPs is 1 m.

### 5.2. Performance Evaluation

**Figure 8:**
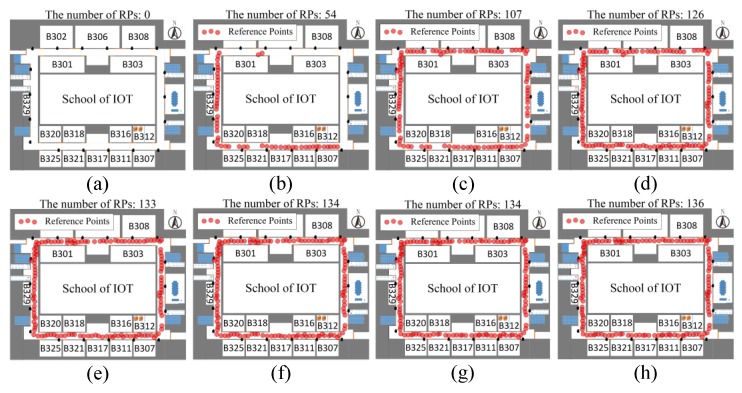
The process of the fingerprint database construction.

Figure 8 depicts the process of the fingerprint database construction. In this experiment, there are some users who moved randomly with a smartphone in the indoor environment. The location and fingerprint data will be continuously collected to our server. From Figure 8 we can find that the number of RP points in the fingerprint database are very few at the beginning. During the initial phase of the UILoc system, the number of reference points increases rapidly, as shown in (a), (b) and (c), until the environment is evenly distributed with reference points. At last, as shown in (g) and (h), the fingerprint database reaches a steady state.

**Figure 9:**
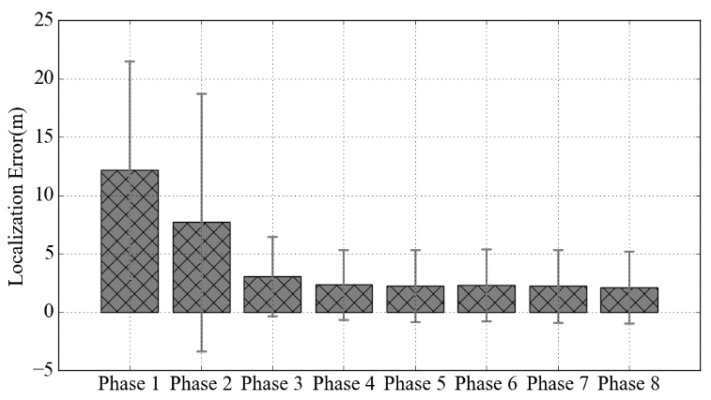
The average localization error and the standard deviation of the initial localization model under different phases.

Figure 9 shows the average localization error and the standard deviation of our initial localization model under 8 different phases corresponding to the 8 graphs in Figure 8. As expected, the accuracy of the initial localization model is very low in phase 1 and phase 2, because the location at this phase is mainly provided by the least squares method. From phase 3 to phase 5, the number of RPs in the database was growing rapidly, so the localization error and the standard deviation are rapidly decreased. At last, the positioning error converges at around 2.15 m.

**Figure 10:**
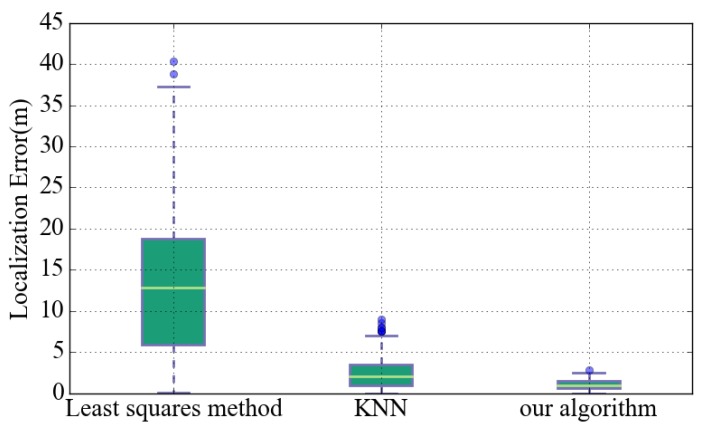
The localization error of different initial localization algorithms.

Figure 10 is based on the localization error of each initial localization algorithm. It can be seen that the error interval of the least squares (LS) algorithm is the largest because of the signal fluctuations. Compared to other algorithms, the number of outliers in our algorithm is the least because the weighted fusion algorithm can suppress the impact of signal fluctuation. In UILoc, our initial localization model can provide a more accurate initial positioning.

In order to analyze the localization error of UILoc, we evaluated UILoc algorithm in a real experimental environment. In the experiment, the user walked in the experimental area with a fixed step length, then we used the PDR; PDR and BLE; PDR, BLE and PF; PDR, BLE and RM (reliable model); and our method (ie UILoc) for trajectory estimation; all of these algorithms are briefly described as PDR, PB, PBP, PBR and UILoc.

**Figure 11:**
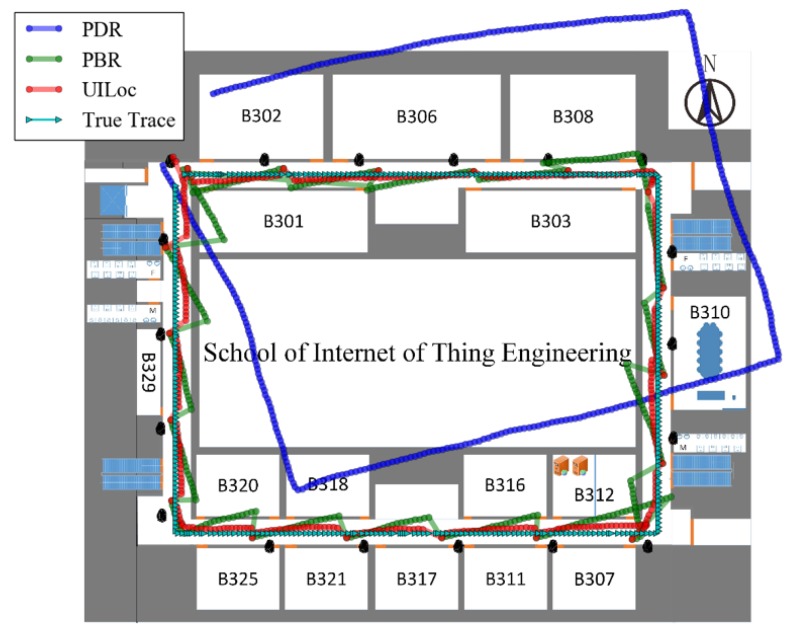
Estimated trajectories with different algorithms.

Figure 11 shows the intuitive trajectory estimations by some algorithms in our experimental area. In the experiment, we compared the PDR, PBR and UILoc. It can be seen that the cumulative error of the PDR is very serious in the absence of the landmarks’ correction, but it is shown that the PDR provides continuous position estimation. As the green track shown, we can use landmarks to restrain the cumulative error of PDR. As shown in the bottom right corner of the Figure 11, the PBR algorithm has the phenomenon of repeated correction. But in UILoc, the reliable model prevents the error caused by repeated correction.

**Figure 12:**
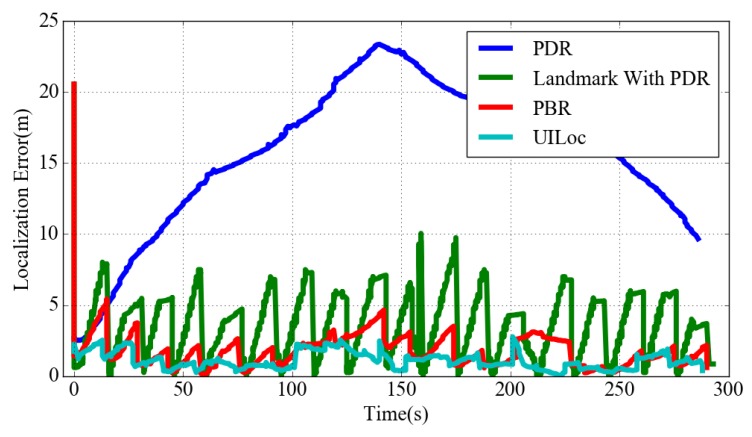
Localization errors of indoor trajectories using different algorithms.

Figure 12 describes the real-time localization errors of indoor trajectories using PDR, PB (landmark with PDR), PBR and UILoc. As can be seen from the figure, without the aid of the reliable model PB’s localization error is significantly larger than the PBR algorithm. As the red line shows, in the initial positioning phase the initial localization error of PBR is much larger than UILoc. So the UILoc system can provide a more stable position estimation.

Table 1 is a summary of localization errors of different algorithms, we also compared the root mean square errors (RMSE) of different algorithms. UILoc’s positioning accuracy has been greatly improved compared to the fingerprint positioning algorithms, e.g., KNN [24], UnLoc [28], Zee [9]. In the KNN algorithm, the mean of the positioning error is 2.36 m; in UnLoc [28], the mean of the positioning error is 2.0 m; in the Zee [9] algorithm, the mean error is about 5 m. Without the reliable model, the positioning error of the PB algorithm is 3.06 m, and after adding the reliable model module, the localization error is reduced to 1.77 m. After adapting the reliable model and the initial localization model to UILoc, the average positioning error is reduced to 1.11 m and the error of 50% samples is kept within 1 m. In this experiment, we also get a maximum positioning error of 2.77 m.

**Figure 13:**
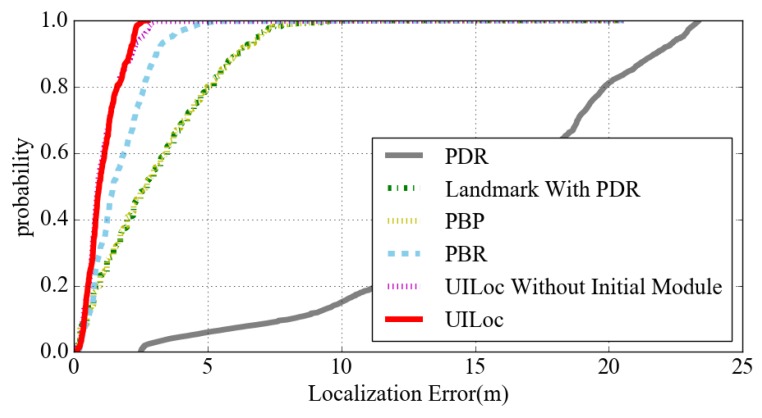
The cumulative probability of location error with different algorithms.

Figure 13 is the cumulative probability graph of each positioning algorithm. Due to the PDR cumulative error, the convergence rate of the PDR is very slow. The convergence rate of PBR is obviously improved after adding the RM module. We can see from the figure that the convergence rate of UILoc is faster than that of UILoc without the initial module. The UILoc can keep the positioning error within the range of 3 m steadily.

Finally, we implemented the UILoc on the Android platform and the reader can view experimental videos on our webpages [35,36].

## 6. Conclusions and Future Work

In this work, we have proposed the UILoc, which builds and updates the fingerprint database automatically. It provides accurate initial position estimation by an initial localization module and provides stable and accurate localization by a reliable model. Firstly, the popular PDR approach has the well-known drift problem. Since iBeacons can provide a high localization accuracy and can be easily deployed in real situations, we corrected the drift of the PDR approach by iBeacons. Since we used the PDR method to provide the basic position estimate, the accuracy of the initial position estimation is very important. We proposed the initial localization module (a weighted fusion algorithm that combined a fingerprint method and least squares method) to estimate a user’s initial location. Lastly, as the popular landmark approach just detects the strongest signal and corrects a user’s position, which may cause the problem of repeated corrections and result in extra localization errors or the instability of localization, we proposed the reliable model which can provide stable and accurate localization. Preliminary experimental results show that UILoc demonstrates low cost, rapid system deployment and high positioning accuracy. Results show that the UILoc achieved an average error of 1.11 m and can provide initial localization with an average error of 2.15 m.

Our ongoing research focuses on making UILoc feasible and pervasive in various applied environments and buildings.

## Figures and Tables

**Table 1:** Summary of localization results.

Algorithm	50%	75%	Mean	RMSE
**PDR**	16.52	19.29	15.56	16.41
**PB**	2.75	4.60	3.06	3.76
**PBP**	2.75	4.50	3.04	3.74
**PBR**	1.49	2.44	1.77	2.26
**KNN**	2.0	3.5	2.36	2.97
**UILoc**	0.96	1.47	1.11	1.26
